# Effects of the Skills4Genius sports-based training program in creative behavior

**DOI:** 10.1371/journal.pone.0172520

**Published:** 2017-02-23

**Authors:** Sara Santos, Sergio Jiménez, Jaime Sampaio, Nuno Leite

**Affiliations:** 1 Research Centre in Sports Sciences, Health Sciences and Human Development, CIDESD, CreativeLab Research Community, University of Trás-os-Montes and Alto Douro, Vila Real, Portugal; 2 Facultad de Ciencias de la Actividad Física y el Deporte, Universidad Europea de Madrid, Madrid, Spain; University e-Campus, ITALY

## Abstract

Team Sports has been suggested as a suitable environment to investigate creative behavior. This study’s purpose was two-fold: first, it intended to identify the effects of the Skills4Genius sports-bases training program in thinking, motor, and in-game creative behavior in team sports. Second, it aimed to investigate the relationship between creative thinking and in-game creativity. Forty children from primary school were allocated into control (n = 18, age: 9.2±0.4) and experimental (n = 22, age: 9.5±0.7) groups. The experimental group participated in a five-month training program involving either creative thinking, diversification, physical literacy, and nonlinear pedagogy approaches (Skills4Genius). Variables in the study included: a) creative thinking; b) motor performance (vertical jump, speed, and agility); c) in-game individual creative behavior (attempts, fluency, and versatility); and d) in-game collective behavior (positional regularity). The results suggested that the Skills4Genius program fostered creative thinking, agility, and speed performance. Moreover, it stretched the in-game individual creative behavior mainly through the improvement of the attempts and versatility of the player’s actions. Lastly, it nurtured a better learning of the tactical principles, whereas the children were more coordinated with their teammates’ and opponents’ positioning. Additionally, this study presents a positive correlation linking creative thinking and in-game creative performance. These findings highlighted that creativity is facilitated while players become more thinking and game-skilled. Coaches and educators may apply this functional environment to inspire children’s disposition to move outside the box and trigger a creative spark in team sports players. Notwithstanding, the sports environment is ideally suited for fostering creative behavior, a higher-order disposition that will go on to differentiate the everyday life of a child.

## Introduction

Creativity in team sports inspires players to achieve remarkable behaviors that contribute to sports upgrade. A creative player must be able to move and attune outside the box under the guidance of the sports environment. Thus, he will be able to solve specific game problems in a novel, feasible, unexpected, and original way by starting a single act or flowing in a collective action that will lead to the team’s success [1]. Despite the complexity of the concept, findings suggest that creativity is a disposition that can be improved through optimizing the environment and developing proper training programs [2–4]. Creative behavior can be reached throughout a long preparatory process, which in the early years prevails the Personal-creative type and thereafter a possible Historical-creative type. The P-type is internal to the player and it is related with the exploration of new behaviors to overcome individual limitations. Otherwise, the H-type is a behavior widely recognized as innovator and usually expressed in the creativity concepts [5]. Currently, there is a clear need for creativity and consequently, it is required to invest in programs that are designed to empower this skill during the lifespan [3].

Nowadays, the environment created for players stifles their creative potential instead of stretching their thinking and technical-tactical variability. To change this prospective, the latest research recommends several tenets that training programs should take into consideration to boost creativity in team sports settings [1]. The Creativity Developmental Framework guidelines proposes a holistic perspective which underpins the following approaches: i) diversified practice; ii) physical literacy (learning the fundamental movement and game skills); iii) nonlinear-pedagogy which encompasses the teaching games for understanding and differential learning and iv) creative thinking (divergent and convergent thinking). The training program assumptions adopted in this study were sustained in the *exploratory* stage of the previous Framework. An integrated contribution of these approaches assure the optimal conditions to nurture the creative behavior [1].

The previous Framework highlights the relationship between the sport participation and the general development of creative thinking outside of the sport context, as well as reinforces the importance of training the general creative thinking as to reach full creative potential in the field. In other words, is it really possible to develop creative thinking through team sports? Will nurturing general creative thinking form open mind players which exploit original possibilities in the game? What are the appropriate contextual conditions that may arouse this disposition? Several findings revealed the positive impact of team sports participation on children’s cognitive functioning [6, 7], but their role on the underlying cognitive mechanisms related with creativity still remains inconclusive. However, it seems that physical exercise enhance divergent and convergent thinking, both considered the main ingredients of creativity [8, 9]. Divergent thinking consists of identifying a range of possibilities to solve problems in an original way [10]. However, divergent thinking should not be misunderstood as being synonymous with creativity. In fact, convergent thinking also plays an important role in the creative process, guiding players towards the single best and most unusual option [9].

An important starting point to bridge the gap of how to nurture a mood of generating novel solutions is to create enabling environments that reward curiosity and exploration, enhance intrinsic motivation, encourage risk taking, give opportunities for choice and discovery, and also nurture self-management skills [11]. In this endeavor, several sports training-related variables are placed in evidence. A growing body of research demonstrated the effectiveness of a diversified training program sustained in deliberate play to develop creative behavior [12]. It seems that the involvement in many different sports/game situations during the early years is mandatory to improve creativity [13]. Further, deliberate play is considered to be fertile ground for the enhancement of creative expressions [14]. The low structured and playful environments provide freedom to explore a broader range of solutions to overcome game problems [15, 16]. Additionally, these open environments tend to display a myriad of divergent ideas in other non-specific domains (i.e., figurative, verbal, writing and arts) [17].

In this regard, it is also recognized that early participation in highly exploratory activities builds a solid foundation of physical literacy [18, 19]. This concept transcends the physical domain, including one’s ability to use cognitive processes, such as reading environmental information and using those cues to respond successfully with intelligence and imagination [20]. In the sport settings, physical literacy is usually associated with the mastery of fundamental motor skills (i.e., speed, agility, jumping) that should be taught in a complementary way with the fundamental sports skills (i.e., learning to kick the ball without an interception of the opposing team) to move competently in all types of environments [21]. In football, for example, the jumps, straight sprints, and the change of directions are the more frequent movements that precede goal-scoring opportunities [22]. However, to make a proper decision, it is essential to identify the environmental cues, such as the opponents’ and teammates’ displacements. Nevertheless, most of the research focuses only on individual aspects of creativity and fully neglects the dynamics of team coordination, because groups can also generate creative ideas together [23]. Therefore, the mastery of both fundamental skills, which are considered the building blocks of sport performance, provide the basic tools to trigger skillful and creative behaviors.

An important feature is that creativity can only be requested if the environment requests creativity. Thus, creativity training programs must be grounded in nonlinearity. A nonlinear pedagogy provides contextual interference conditions for training functional and novel solutions within a set of specific task constraints [24–26]. Commonly, these types of constraints can be readily manipulated to boost the exploratory behavior and unlock the player’s divergent thinking [27, 28]. Several nonlinear principles are usually incorporated in game-centred pedagogies, such as the Teaching Games for Understanding (TGfU) approach [29–31]. The TGfU interacting environment provides multi-stability, which means more options for players to make decisions, which requires a broader attentional demand, and in turn, assures an adequate development of the breadth of attention [31, 32]. A wide breadth of attention allows players to better attune and perceive environment cues in order to pursue and generate novel solutions [33]. Still, the key pedagogical principles of nonlinear pedagogy leads to higher levels of intrinsic motivation a crucial assumption to maintain the children committed in sports [34].

For creativity training, a nonlinear intervention should be coupled with the differential learning method, which focuses on the movement variability that occurs without movement repetitions or corrections during the skill acquisition process [35, 36]. This approach is considered nonlinear because it encourages children to explore the adaptive mechanisms through applying infinite variations in technique movements to ready them for dealing with disturbances in game settings [37, 38]. The available research has revealed that successful learners demonstrated several instances of kicking pattern variability and higher exploration during the training sessions [39]. Creativity is strengthened by the improvement of the movement variability, which facilitates the player’s technical-tactical exploration. This differential preparation is filled with adversity and allows a better reorganization of the knowledgeable skills and the learning of new patterns to produce novel movements and tactical arrangements [38]. Possibly, this functional climate underpinned in the training program serves as a precursor for creative actions, since a child tends to pursue several alternatives to solve a problem using unconventional methods.

Creativity research related to sport has focused specially on understanding the impact of particular training programs only on the enhancement of sport-specific creativity. In fact, the combined influence of creative thinking, diversification, physical literacy and nonlinear pedagogies in a single training program remains unexplored. However, it is also crucial to comprise the effects of this type of programs on the general creative thinking. To address these issues, the aim of this study was two-fold: firstly, it intends to identify the effects of the Skills4Genius sports-based training program in creative thinking, motor skills (vertical jump, speed, and agility), in-game individual creative behavior (exploratory behavior), and in-game collective behavior (positional regularity) in team sports. Secondly, it explores the possible relationship between creative thinking and in-game creativity.

## Methods

### Participants

A total of 40 children from primary school (third and fourth grades), were randomly distributed across control and experimental groups. The control group was composed of 18 children (pre-test age: 9.2±0.4 years; height: 132.0±4.7 cm; weight: 30.3±5.7 kg; body mass index: 17.3±2.4; body fat: 19.3±7.6) and post-test (age: 9.4±0.6 years; height: 133.2±4.8 cm; weight: 40.0±5.9 kg; body mass index: 18.3±3.3; body fat: 20.9±10.5). The experimental group was comprised of 22 children (age: 9.5±0.7 years; height: 136.7±7.4 cm; weight: 34.5±7.0 kg; body mass index: 18.3±2.2; body fat: 19.4±6.7) and post-test (age: 9.6±0.6 years; height: 137.1±7.5 cm; weight: 34.8±6.1 kg; body mass index: 18.6±2.1; body fat: 19.6±6.7), who participated in the training program. The initial sample comprised 25 children for each experimental and control groups, however a few of them withdraw throughout the 5 months due to a variety of reasons, while others have not fulfilled with the requirements originally defined related to the attendance in the training program. The Skills4Genius training program was applied as extracurricular school activity. Further, both groups until the end of the program had not had previous experiences in structured practice in a club and only participated in regular physical education classes at school. Additionally, the participants did not attend any enrichment activity (such as, music, language or arts extra activities) before and during the training program. Players and their parents were fully informed of the aims and procedures of the study and signed a mandatory consent form to participate. All participants were notified that they could withdraw from the study at any time. The study protocol was approved and followed the guidelines stated by the Ethics Committee of the Research Centre for Sport Sciences, Health and Human Development, based at Vila Real (Portugal) and conformed to the recommendations of the Declaration of Helsinki.

## Instruments

### Creative thinking testing

The participants’ creative thinking was measured through the Torrance Test of Creative Thinking—Figural Version: form A. The Torrance Test of Creative Thinking has been translated into over 35 languages and is widely recognized and referenced as a valid and reliable instrument of creative thinking assessment [40]. The Torrance Test of Creative Thinking—Figural was comprised of the following three activities, all of which required unusual drawings that have standard shapes as parts: a) draw a picture, using a pear or jelly bean shape provided on the page as a stimulus; b) finish a drawing, using ten incomplete figures to make a completed object or picture; and c) compose a different drawing using parallel lines, where children used three pages of lines to create pictures [41]. Moreover, each drawing was scored using five subscales: fluency; elaboration; originality; resistance to premature closure (closure); abstractness of titles (titles); and also 13 creative personality traits that comprise the students’ creative strengths. Fluency is the ability to produce a large number of figural images. Elaboration reflects the participant’s ability to apply details in order to extend an idea. Originality represents the ability to produce unusual, unexpected, yet relevant ideas as determined by statistical infrequency. Resistance to premature closure is based on the conclusion that creative behavior requires a person to keep an open mind while processing information. Abstractness of titles is based on the idea that creativity requires an abstraction of thought, and measures the degree in which a title moves beyond concrete labeling of the pictures drawn [41]. The sum of these five norm-referenced scores make up the creativity score, which is an indicator of creative potential.

### Motor performance

The vertical jump ability was assessed through a free arms countermovement jump using the optojump system (Microgate SARL, Italy), in order to determine the explosive power of the lower limb. This test involved the participants lowering themselves from an upright standing position until approximating a knee angle of 90 degrees, followed immediately by a vertical jump as high as possible. They were allowed to swing their arms freely. Moreover, the anaerobic performance was evaluated using a straight sprint test. Participants were instructed to run as fast as possible along the 15m distance from a standing-start position. The agility assessment was evaluated through a modified agility T-test and its reliability was outlined by Sassi and colleagues [42]. The modified agility T-test preserved the same nature of displacement of the T-test, except for the measures of intercone distance that was reduced to be more representative of team sports requirements. The participants started each trail from a standing position 0.5m behind the sensor with both of their feet behind the starting point. On command, the participants: 1) sprinted 5m forward to the center cone and touched the top of it with their right hand; 2) sidestepped 2.5m to the left cone and touched its top with the left hand; 3) sidestepped 5m to the far right cone; 4) sidestepped back to the left to the center cone; and then 5) ran 5m backward to the finish line. The participants repeated the test if they failed to run the course as instructed. The modified agility T-test and 15m straight sprint performances were measured using a witty photocell system (Microgate, Bolzano, Italy).

### In-game creative behavior

#### Individual behavior (technical performance)

The small-sided games were recorded using two digital cameras, both Sony NV-GS230, from a fixed position. Then, the individual creative behavior was assessed through a computerized notational analysis using Simi Scout software (version 2.0.0.174, Simi Reality Motion Systems GmbH, 2007, Unterschleissheim, Germany). To evaluate the offensive technical creativity in ball possession, the data provided by the Simi Scout Software was organized in a designed Excel for Windows^®^ spreadsheet entitled Creativity Behavior Assessment in Team Sports (CBATS). Measurements included pass, dribble and shot and the examination of participants’ actions was divided into successful and unsuccessful [43]. Further, the previous technical-tactical variables were classified according to creativity components, which was derived from creativity research and adapted to the sport contexts [1]. The creativity components assessed established a creative behavior score: a) attempts, recognized as any effort to perform different actions, even in unsuccessful movements; b) fluency, or the ability to execute as many successful movement actions as possible; and c) versatility, which was identified as the ability to generate a diversity of actions, such as executing different forms to pass or shoot. Thus, attempts were considered the unsuccessful actions, while the fluency and versatility comprised the successful actions. A detailed description of the variables observed using the CBATS are presented in the S1 Table. The CBTAS enabled us to automatically carry out the calculations of the individual technical-tactical variables (attempts, fluency, and versatility) to determine a final score. The S2 Table provides a simplified example of the CBATS’ structure. Thus, the sum of fluent actions for the pass, dribble and shot result in the total score of fluency and the same process was occurred with attempts and versatility. Moreover, the previous variables were assessed considering absolute values of occurrence (frequency). Additionally, the present study address the exploratory behavior as a concept that combine the attempts, fluency and versatile actions. An experienced sport performance analyst gathered the data and reliability was inspected by retesting 17.5% of the sample one week later, calculating the attempts, fluency and versatility for the pass, dribble and shot. The obtained intra-rater reliability coefficients were high (ICC > 0.83) [44].

#### Collective behavior (tactical performance)

Players’ displacements were captured using Global Positioning System devices—GPS and computed using dedicated routines in Matlab R2013b software (version 6.5; Math-Works, Inc., Massachusetts, USA) according to the guidelines suggested by Folgado and Colleagues [45]. The data obtained was used to calculate the distance to team centroid and the distance to opponent team centroid. These positional-derived variables were processed with an approximate entropy technique—ApEn [46, 47] to compute the amount of players’ movement regularity of the time-series corresponding to the distance between players. The file exchange to computing approximate entropy for a time-series data it is presented in the S1 File. The values range between 0 (higher regularity) and 2 (lower regularity) of unitless real numbers. Lower values means more regular, repeatable and less chaotic sequences in players’ movements. In a practical sense, these processing techniques help us to better understand if the players’ positioning decisions replicate a regular movement pattern in relation to the teammates and opponents. Thus, ApEn is useful to understanding the regularity and predictability of a positional movement [48].

## Procedures

Anthropometric measurements such as weight, height, body mass index and body fat percentage, took place before the pre-test session. Also before the pre-test measurements participants were familiarized with the tests. All participants were tested before (pre-test) and after (post-test) the 5 months training program period. The data collection was divided into 3 different days. In first one, the creative thinking was measured in a classroom. The researchers provided a brief explanation of the Torrance Test of Creative Thinking and its completion was conducted in a quiet environment, lasting for 30 minutes. Ten minutes were required to complete each activity. As recommended [41], before the administration of the Torrance Test of Creative Thinking—Figural the participants performed a warm-up activity based on a brainstorming to arouse their creative incubation. Still, the researchers stressed that the participants could enjoy and view the tests as a series of pleasurable activities. In the second session, the researchers collected the motor performance variables. Before the assessment, the participants completed a 15-minute standardized warm-up, including jogging, bilateral movements, dynamic stretching, skipping, jumping, and recreational games. First, was measured the vertical jump, then the straight sprint and finally the agility. In all previous tests, two trials were performed and the best performance of each was considered. All participants did not receive verbal encouragement. The third session was performed to measure the in-game variables. Before the testing session, there was a standardized 15-min warm-up based in ball possession games without targets. The individual and collective game behavior was assessed during a 3-a-side, small-sided football game with goalkeeper (GK+3x3+GK) in artificial turf on a 10x12m (length x width) pitch. The small-sided game protocol was composed by two bouts of 4-minute-durations interspersed with a 2 minutes of passive recovery. All small-sided games were performed as much as possible with the official game rules, except the use of offside rule. Several balls were placed around the field to ensure the replacement as fast as possible. The coach minimized his intervention and did not provide feedback during the game.

## Skills4Genius training program design

The Skills4Genius training program lasted for 5 months with three sessions per week with a duration of 60 minutes each (for a total of 60 sessions, or about 12 per month). To ensure an appropriate enrichment environment, the program was supported with the following assumptions: a) creative thinking training; b) diversification; c) physical literacy; and lastly d) nonlinear pedagogy that accounts with the TGfU and differential learning approaches (see Table 1).

**Table 1:** Description of the general Skills4genius training program tenets.

Tenets of the Skills4Genius Training Program
**Creative Environment–*Unlimited thinking*, *unlimited performance***
For children it is extremely important to explore without limits in several domains. Therefore, in the initial part of the training sessions, children had a book with creativity tasks. These activities were comprised of motor, figural, written, and verbal tasks that encompassed divergent and convergent abilities (i.e., creating their own dribble and technical skills; completing drawings; deciding what they can do with a specific object; writing a letter from the moon; or solving critically a problem in different ways). Creative tasks were executed in individual forms, in pairs, or in groups, depending on the program timeline. This enrichment environment may arouse the creative thinking incubation and the children’s disposition to attempt and diverse new solutions during the remaining sport session.
**Diversification—*Diversify to create in any situation***
The training program encompasses the learning of three main sports: handball, football and basketball. In each week, the children practiced one of the chosen sports, except on a random day per week in which they practiced a different sport (i.e., frisbee, field hockey, volleyball, rugby, and others). At the end of training sessions, the children always played a formal game, which was intended to ensure the sport’s official rules. However, in the last three months of the enrichment program, the children often played an adapted version of Kronum game called Game4Genius. These activities allowed them to play more than one type of sport simultaneously (i.e., football mixed with handball). Also, the challenges and activities applied in the program improves technical-tactical basic elements through the incorporation of several types of balls and materials and use at the same time different parts of body to play, generating high impressibility and attentional demand.
**Physical Literacy—*Equip children with basics***
In order to achieve the children’s creative potential, the training program states that physical literacy mastery is mandatory. Therefore, in all training sessions the fundamental motor skills (agility, speed, coordination, and plyometric training) are developed through circuits always embodied in representative contexts. Otherwise, the fundamental game skills are enhanced through ludic games, which encompasses the fundamental learning game principles proposed by Memmert and Harvey [65] (i.e., dribbling attempts, support and orienting, taking the ball near the goal, attacking the goal, playing together, achieving advantages, and using gaps and defense, which involves defending the goal, gaining the ball, and defending attacking players). As previously mentioned, the training program was always supported by an environment fulfilled by encouragement of creativity training components (attempts, fluency, and versatile actions) incorporated in the training of the fundamental game principles.
**Nonlinear Pedagogy—*Adapt*, *explore*, *and create without limits***
The training program focused on the manipulation of constraints and game representativeness, which encourages the emergence of functional and novel movement solutions to overcome a game challenge. For that, appropriate contextual interference is necessary. To avoid children becoming overwhelmed in the earlier stages of skill acquisition and due to their lack of sports experiences, a moderate contextual interference was chosen [see 54]. With a nonlinear pedagogy in mind, the tasks combine different skills during a single drill.
**- Teaching Games for Understanding**
In fact, the training sessions implement several forms of small-sided games that promote exploratory behavior and unlock the children’s creative potential. Furthermore, simple constraints were applied, such as: using a small number of players in unbalanced formats; playing in numerical advantage or in equal number of players (i.e., 1vs1, 2vs1, 2vs2, 3vs1); reducing the playing area; and/or adding several targets. These constraints create multiple 1-on-1 duels that increase the number of ball contacts per player and their perceptual responses. Still, several moderate constraints are used, like increasing the numerical duels (3vs3), as well as playing in numerical disadvantage (2vs3 and 3vs4) and constraining the ball and type of touches to explore team-related movements.
**- Differential learning**
During the program sessions the children had to execute several variations in technique movement (i.e., pass, shoot, or feint) to make them ready to deal with game disturbances (i.e., eyes closed, arm behind the body, arm straight up, rotate arm, do math, juggling a balloon, play with two balls, use different types and sizes of balls, and play with the non-dominant limb). This noisy environment allows children to improvise new behavioral patterns and prevent standardized actions, providing a fundamental basis for motor learning [36].

**Fig 1:**
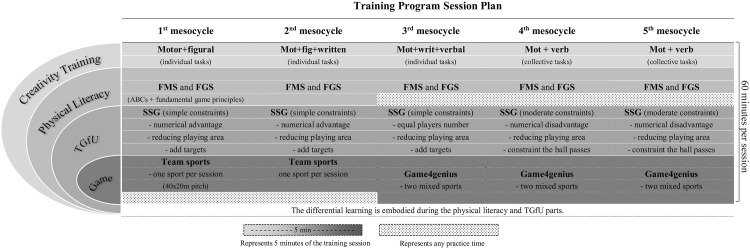
Creativity training program schedule during different mesocycles. Legend: mot., motor; fig.; figural: writ., written; verb., verbal; FMS, fundamental movement skills; FGS, fundamental game skills; ABCs, agility, balance, coordination and speed; TGfU, teaching games for understanding; SSG, small-sided games. A mesocycle corresponds to a month of training which includes 12 practical sessions with 60 minutes each.

Additionally, Fig 1 describes an example of the Skills4Genius training session’s changes during the intervention. Under these practice conditions, the training sessions could be characterized by a large unpredictability and adaptation demands to enhance the functionality of movements and the breadth of attention.

## Statistical analyses

All pre- and post-test values were compared using a magnitude-based inference approach expressed in percept units with 90% confidence limits [49]. The creative thinking, motor, and in-game variables were log-transformed and the minimum difference was defined as 0.2 among participants’ standard deviations. However, a descriptive analysis was performed using mean and standard deviations for each variable (the mean shown is the back-transformed mean of the log transform). Standardized thresholds for Effect Size statistics were considered as follows: 0.2 was trivial; 0.6 was small; 1.2 was moderate; 2.0 was large; and above 2.0 were very large changes and determined to examine the magnitude of differences [49]. Using a customized spreadsheet, it was calculated the probability that the true differences were substantial or trivial [50]. Differences were defined as unclear when the confidence limits for the Effect Size included both substantial positive and negative values (± 0.2*standardisation). In a clear interpretation, the following probabilistic terms were adopted: < 0.5%, most unlikely; 0.5–5%, very unlikely; 5–25%, unlikely; 25–75%, possibly; 75–95%, likely; 95–99.5%, very likely; and >99.5%, most likely [24]. To verify the correlation between the results obtained in thinking and game scores, the Pearson correlation test was used. These data were analyzed using the software package SPSS Statistics 20.0 (SPSS Inc., Chicago, IL, USA). To complement these results the magnitude of correlation [r (90% CL)] between test measures were assessed with previous probabilistic terms. If the 90% CL overlapped positive and negative values, the magnitude was deemed unclear, otherwise the magnitude was deemed to be the observed magnitude [49].

## Results

**Fig 2:**
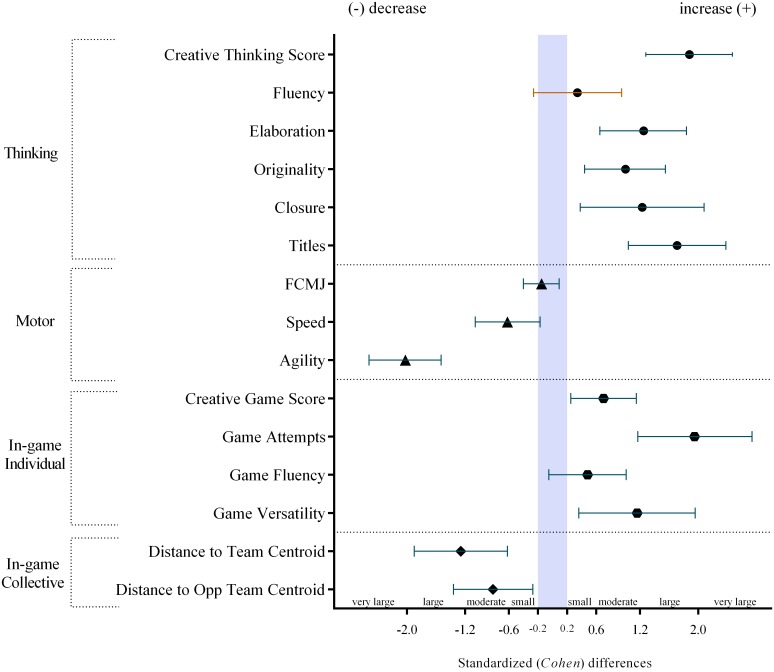
Standardized (Cohen) differences in thinking, motor, in-game individual and in-game collective variables between pre-post test variations. The (+) and (-) symbols represents if the considered variable increase/decrease from pre- to post-test. The blue area represents trivial differences.

The results presented in Table 2 and Fig 2 will be discussed simultaneously. Regarding the creative thinking, the results demonstrate that the experimental group has benefited from a most likely increase (difference in means, %; ±90% confidence limits: 235%; ±131.8%) with a large effect. This increase was mostly expressed in the elaboration (66.3%; ±40.7%), abstractness of titles (389%; ±324.6%) and resistance to premature closure (53.7%; ±46.4%) all with a large effect. While the originality presents a moderate (69.4%; ±50.4%) effect. However, the fluency score presented an unclear trend (13.1%; ±24.7%) due to the slight increase demonstrated in both groups. Still, an interesting finding was the decrease in creative thinking in the control group (pre-test, mean±SD: 9.7; ±4.7, post-test: 4.3; ±2.7).

**Table 2:** Descriptive analysis and standardized (Cohen) differences in group means for the Skills4Genius program effects in thinking, motor and in-game performance between control and experimental groups.

Variables		Control		Experimental		Difference in means: %; ± 90% CL (uncertainty in true differences)
		Pre (mean±SD)	Post (mean±SD)	Pre (mean±SD)	Post (mean±SD)	
Creative Thinking	Creative Thinking Score	9.7±4.7	4.3±2.7	7.8±4.0	12.2±3.9	235.0; ±131.8 (most likely ↑)
	Fluency	21.7±5.6	24.1±9.6	22.8±8.2	26.8±7.1	13.1; ±24.7 (unclear)
	Elaboration	8.2±3.0	5.7±2.0	7.5±2.8	8.9±3.4	66.3; ±40.7 (most likely ↑)
	Originality	10.4±4.2	11.7±4.7	10.4±4.8	18.2±7.8	69.4; ±50.4 (very likely ↑)
	Closure	11.8±3.0	9.4±4.4	12.0±4.2	15.0±4.1	53.7; ±46.4 (very likely ↑)
	Titles	4.2±3.7	3.4±5.1	4.7±4.3	11.5±5.6	389.0; ±324.6 (most likely ↑)
Motor	Free countermovement jump	18.4±4.8	19.8±4.9	24.3±5.6	25.5±3.8	-1.3; ±6.8 (likely trivial↓)
	Speed	3.3±0.2	3.2±0.2	3.1±0.2	3.0±0.3	-4.1; ±2.9 (likely ↓)
	Agility	8.9±0.6	9.5±0.9	8.4±0.8	7.4±0.8	-17.7; ±3.9 (most likely ↓)
In-game individual	Creative Game Score	9.0±4.5	11.1±4.5	14.1±9.0	26.1±11.9	62.0; ±50.9 (very likely ↑)
	Game Attempts	0.8±0.5	0.9±0.5	1.1±0.6	4.8±2.7	359.7; ±299.9 (most likely ↑)
	Game Fluency	19.8±14.6	24.9±16.2	39.5±27.4	69.9±36.4	53.7; ±75.2 (likely ↑)
	Game Versatility	6.2±3.0	7.5±6.3	1.4±0.6	3.3±1.6	182.3; ±220.1 (very likely ↑)
In-game collective	Distance to Team Centroid	0.49±0.1	0.60±0.1	0.68±0.1	0.62±0.1	-25.1; ±11.0 (very likely ↓)
	Distance to Opponent Team Centroid	0.75±0.1	0.82±0.1	0.91±0.1	0.87±0.1	-12.3; ±7.7 (very likely ↓)

Note: CL = confidence limits. ↑ = increase; ↓ = decrease.

Results revealed that the training program was not very effective in the vertical jump enhancement. Comparing both groups’ variations (from pre- to post-test), the experimental group showed a likely trivial trend (-1.3%; ±6.8%). Otherwise, compared to the control group, the training program presented a greater improvement trend in the sprint as well as in the agility performance (sprint: -4.1%; ±2.9% and T-test: -17.7%; ±3.9%) with a moderate and a very large effect, respectively. Despite this, the speed and agility values can be found on the left side of Fig 2, and serve as an indicator of improvement (these values are interpreted in the opposite way). Moreover, compared to the control group, the experimental group presented a very likely 62.0%; ±50.9% (moderate) improvement in the individual creativity game score. These results may be complemented with the attempts outcome (most likely ~360% increase, or very large), versatility of their actions (a very likely ~182% increase, moderate) and fluency (likely ~53% increase, small). The results from both distance to team centroid and distance to opponent team centroid presented a similar trend. While the experimental group decreased their ApEn (indicating higher regularity), the control group increased their ApEn values (decrease the regularity) for both variables. Comparing both groups’ variations, the experimental group showed a very likely decrease trend in distance to team centroid (-25.1%; ±11.0%, large) and also in the distance to opponent team centroid (-12.3%; ±7.7%, moderate).

Finally, the pre-test results of the control and experimental groups indicated a very strong correlation (r; ±90% confidence limits: 0.91; ±0.08 and 0.94; ±0.05, respectively) between thinking and in-game creativity score with a most likely beneficial trend. However, in the post-test measurements, both correlation values decreased (0.45; ±0.33; 0.62; ±0.23). This trend is more accentuated in the control group with a likely positive trend, whereas the experimental group maintains a most likely tendency (Table 3).

**Table 3:** Inferential statistics of thinking and game pre-post variation and the coefficient correlation between pre-pre and pre-post of the control and experimental groups.

Variables	Control			Experimental		
	Δ% (pre-post)	r; ±90% CL pre thinking-pre game (inference)	r; ±90% CL post thinking-post game (inference)	Δ% (pre-post)	r; ±90% CL pre thinking-pre game (inference)	r; ±90% CL post thinking-post game (inference)
Thinking Score	-50.3%	0.91; ±0.08 (most likely positive)	0.45; ±0.33 (likely positive)	66.6%	0.94; ±0.05 (most likely positive)	0.62; ±0.23 (most likely positive)
Game Score	27.4%			106.5%		

**Note:** CL = Confidence limits. A correlation ≥ 0.70 represents a very strong positive relationship; 0.69 to 0.40 indicates a strong positive relationship.

## Discussion

This study aimed to examine the Skills4Genius training program effects in thinking, motor, and in-game (individual and collective) creative behavior in team sports and investigated a possible relationship between creative thinking and sport-specific creativity. Results generally supported the hypothesis that a training program sustained in creative thinking, diversified practice, physical literacy, and nonlinear pedagogy facilitates the development of overall creativity.

An in-depth analysis of the results confirmed that the Skills4Genius training program brought about an improvement in the creative thinking score. This increase was mostly expressed in elaboration, originality, closure, and titles. Indeed, a special feature of the program was to introduce divergent and convergent thinking tasks in the initial part of the training session. Possibly, these non-sports introductory activities may have played an important role in the creative thinking improvement. As indicated in the meta-analysis performed by Ma [4], the program effects were greater as more expressive modalities were trained. On the other hand, the impact of the training program was not equally great on all of the study’s creative components. The program proved to be less sensitive in the fluency enhancement. Our results demonstrated the same slight increase of the fluency in control and experimental groups. A similar trend was presented in Kim’s study [51], verifying that fluency scores without training increased up to third grade and remained static in the fourth grade. In this sense, children who are able to generate a larger quantity of ideas are likely to be more original, which might indicate that during these ages, issues like accuracy and appropriateness of children’s responses must be highlighted. Furthermore, findings on the creative thinking variation across childhood have been ambivalent [51]. Thereby, the control group results indicated a fourth grade slump in Portuguese creative thinking score. This decrease was mostly expressed in the elaboration, closure, and titles. In a practical view, the children’s ability to think in detail, to be intellectually curious and open-minded, as well as their ability to think abstractly and capture the essence of the information involved is compromised during the ages of 9 and 10. The issues that trigger these variations remain unexplored, but the standardized learning method used in the Portuguese regular school may have had a determinant role [11, 52].

In team sports, the physical literacy mastery is mandatory to put into field what goes on in a creative player’s mind. Indeed, the domain of the fundamental motor skills proved useful to enhance a game-related performance [16, 22]. In this regard, the Skills4Genius training program revealed their effectiveness in speed and agility development. These outcomes can be explained by the moderate contextual interference induced by the enrichment program [53, 54]. Further, it is important to stress that the program adopted pre-planned activities and small-sided games formats derived from the TGfU approach. Our results are in line with Chaouachi and colleagues’ standpoint [55], which refers to cumulative effects of combining both types of training having a positive impact in speed and agility enhancement. However, agility presented a better improvement trend. Recently, it was pointed out that training grounded in small-sided games forms is an alternative for agility development [55–58]. Otherwise, the training program was not effective in the vertical jump improvement, even with plyometric tasks [59, 60]. Possibly, low doses of concentrated plyometric training (focused in vertical and horizontal force vectors) provided by pre-planned activities are not enough to induce any adaptation. Moreover, studies report that children from the ages of 9 to 10 years old are not in an optimal window of trainability [61].

Additionally, the Skills4Genius training program encouraged children to expand their sports participation to more game-related behaviors. It is well-known that particularly in team sports, players are required to simultaneously perceive relevant game-information (such as the positions and movements of teammates and opponents), and then deliberately explore a novel action [1]. Our results confirmed that the program enhanced the children’s individual exploratory behavior during the game, through the improvement of their attempts, fluency, and versatility. Note that children’s efforts to diversify their technical-tactical skills during the game, even unsuccessfully (versatility and attempts), were the main components stressed by the program. This was tested instead of the stretching of their standardized ability to respond successfully (fluency). In fact, the mastery of the fundamental sport skills allows players to explore and improvise without limits, which is a precondition of player’s creativity [27, 28, 62]. Conversely to the technical-tactical variability presented in individual performance, the distance to team centroid and distance to opponent team centroid, both positional variables revealed a high regularity (more repeatable movement patterns), indicating the emergence of functional and adaptive movements in the experimental group. It seems that the training program nurtures a better learning about fundamental tactical principles related to space occupation, whereas the children were more coordinated with their teammates’ and opponents’ positioning. This intentional positional adjustment occurred as a result of the players’ game perceptions, revealing a better understanding of the game [46]. Team sports creativity is extremely dependent on the interaction of all elements (teammates and opponents), which leads to the shared creation processes. For that, players should develop a consciousness of the environment changes to dynamically and continually coordinate their actions [23]. Therefore, the technical variability did not impair the game regularity, in turn, it seems that positional adjustments were facilitated while players become more skilled.

In this vein, children will be better prepared to read the game and move outside the box, and ultimately to boost the probability of a creative behavior emergence. Possibly, the non-sport initial activities arouse creative thinking incubation and inspire the children’s disposition to explore new solutions during the remaining sporting session. Moreover, it could be speculated that variability demands provided by diversification, embodied in nonlinearity, nurtures great effects in children’s capacity to adjust motor actions and encourages players to unlock their creative potential. Both types of practice provide freedom to experiment and produce a variety of novel motion configurations, which in turn, gives the children the opportunity to explore and adapt continuously [14, 26, 31, 37]. The game situations presented at the end of the training session (i.e., games simultaneously played with hands and feet, like handball coupled with football) are an accurate example of how to promote variability and unpredictable demands. The *noise* provided by the task constraints manipulation in these game-centred approaches, also promotes the exploratory behavior [29, 63]. Still, these ecological settings allow children to become more proficient at perceiving environment cues and constant changes in game situations, while learning the fundamental tactical principles, explaining in part, the reported trend in the collective positional behavior. Undoubtedly, children should be encouraged to develop game understanding and tactical consciousness [31]. Notwithstanding, children’s perceptual and action adaptability seems to be provided by the improvement in the breadth of attention. Players need a wide breadth of attention in order to generate tactical behavior and seek for different solutions [32]. The enhancement of the ability to attune the relevant information in team sports was also observed in an earlier creativity program [12]. Indeed, the creativity development is facilitated while players become more skilled and have acquire better attentional abilities [64].

Lastly, a positive correlation was found between thinking and in-game individual creativity score, indicating that the unlimited thinking was related with creative game performance. In the pre-test measurements this correlation was very strong, clearly revealing a positive interdependence. Nevertheless, the correlation decreased after the training program application or 5 months later. Apparently, the step decline in the creative thinking and the continuously improvement in the sport-specific creativity explains the higher variation in the control group correlation. Indeed, at this age a slump in the creative thinking occurs. However this tendency was not verified in the game performance. While the creative thinking was not stimulated, instead the fundamental game skills increased by the physical education commitment creating an unevenness between both variables. On the other hand, despite the thinking and game increasing in the experimental group, the correlation slightly decreased. This trend could be explained by the unbalanced thinking and game development. As expected, children improved more game skills rather than thinking abilities, which was not surprising since the enrichment program structure was essentially composed of sport activities. In fact, these results suggest that it is also important to nurture thinking abilities in sports training programs to form open-minded players who exploit all available creative possibilities in the field. As well as, avoided the potential creativity decreased with age. According to the previous connections, the program also provided a lack of improvement in the fluency scores, either in thinking and in-game creativity. This trend may indicates a strong relation between thinking and game creative behavior, since exactly the same components were less stressed. Thus, in forthcoming training programs this issue should assume more attention. Surely, further research is required in this domain to understand the variation in the creative thinking and sport-specific creativity during the life course, and to what extent they complement each other. Further, the impact of enrichment programs in distinct age groups should be considered. Additionally, it is important to overcome a few limitations related with the homogeneity of groups and also carefully control the developmental level of the participants in several domains (maturation, cognition and motor skills). Overall, this insightful study recognizes that environmental interference induced by the training program, can be used to optimize the thinking, motor, and in-game performance in children without previous experience in sports, supporting the development of players’ creativity. Under these practice conditions, the program fosters skillfulness behaviors that play an important role in triggering a creative spark in team sports players.

## Conclusions

In summary, this study demonstrated that a training program sustained in creative thinking, diversified practice, physical literacy, and nonlinear pedagogy has a great potential for fostering overall creativity in team sports. In this sense, the central tenets of the program leads to an enrichment in creative thinking and improves fundamental motor skills, such as agility and speed. Moreover, the improvement of fundamental game skills was observed through the exploratory behavior boost of individual technical actions. The training program nurtures a better learning about fundamental tactical principles related with space occupation, promoting a consciousness of the environmental changes. Additionally, this study encompasses the first report linking creative thinking and in-game creative performance, demonstrating how important is to nurture non-sport creative thinking concomitantly with a technical-tactical sports mastery. Our findings have several implications for coaching and teaching pedagogies. This type of enrichment program could be easily integrated into the Physical Education curriculum as creativity continues to be underdeveloped in schools and sports structures. To finish a range of meaningful practical implications should be highlighted. These include:

- Coaches and educators may apply an enrichment training to inspire children’s disposition to think and move outside the box.
- A sport environment is ideally suited for fostering creative behavior and help to avoid the decrease of creativity with age.
- The training program nurtures the fundamental motor skills and prepare children to read the game, as well as explore unusual technical-tactical behaviors.
- The interdependence between creative thinking and sport-specific creativity indicates that both should be trained as complementary pairs. Thus, the unlimited thinking leads to creative executions in the field, as well as the opposite.

## Supporting information

S1 File File exchange to computing approximate entropy for a time-series data. (DOCX)

S1 Table Description of the variables observed using the Creative Behavior Assessment in Team Sports—CBATS. (DOCX)

S2 Table A simplified example of the measurement the in-game technical skills of one player using the Creative Behavior Assessment in Team Sports—CBATS. (DOCX)
