# Supplementary material for: Effects of the Skills4Genius sports-based training program in creative behavior
Source: PLoS One. 2017 Feb 23;12(2):e0172520. doi: 10.1371/journal.pone.0172520 (PMC5322953; doi:10.1371/journal.pone.0172520)
Supplement: S1 File — (DOCX) [file pone.0172520.s001.docx]

**S1 File**

This appendix provides the file exchange to computing approximate entropy for a time-series data (Copyright (c) 2010, Avinash Parnandi).

<https://www.mathworks.com/matlabcentral/fileexchange/26546-approximate-entropy?requestedDomain=www.mathworks.com>
