# Supplementary material for: Effects of the Skills4Genius sports-based training program in creative behavior
Source: PLoS One. 2017 Feb 23;12(2):e0172520. doi: 10.1371/journal.pone.0172520 (PMC5322953; doi:10.1371/journal.pone.0172520)
Supplement: S1 Table — (DOCX) [file pone.0172520.s002.docx]

**Supporting Information**

**S1 Table**

This appendix (S1 table) describes the meaning of unsuccessful/successful variables; standardized/non-standardized actions as well as, the creative components embodied in technical skills (i.e., attempts, fluency and versatility of pass, dribble and shot), associated to the Creative Behavior Assessment in Team Sports (CBATS).

**S1 Table.** Description of the variables observed using the Creative Behavior Assessment in Team Sports – CBATS.

| **Variables** | | **Description** |
| --- | --- | --- |
| Pass | successful | Intentional played ball from one player to a teammate. |
|  | unsuccessful | Pass that does not reach the intended teammate (i.e., ball interception by the opponent team). |
|  | attempts | When the pass does not reach the receiver, but the player tried to explore a non-standardized action. |
|  | fluency | When the pass reached the receiver but in a standardized way (it is effective). |
|  | versatility | When the pass reached the receiver but in a non-standardized way (effective and different). |
|  | standardized behavior | When a player performed a pass with the dominant limb and using the inside part of the foot. |
|  | non-standardized behavior | Consider all executions that are different from the standardized pass (i.e., pass with the non-dominant limb, lift the ball above the opponents, pass with head, back pass, touch inside and pass, heel pass, and others). |
| Dribble | successful | Initiative by the player with the ball to explore a duel against his direct opponent or opponents in order to gain space to perform a pass or score, while retaining the possession of the ball. |
|  | unsuccessful | Player in possession of the ball failed to overcome the defending player (i.e., dribbler is tackled). |
|  | attempts | Player that failed to progress in the pitch or gain a favorable position, when realized a non-standardized dribble. |
|  | fluency | Player dribbled the opponent and progressed in the pitch or moved towards a more favorable position using a standardized dribble (i.e., side touch, double scissors, side step). |
|  | versatility | Player dribbled the opponent and progressed in the pitch or moved towards a more favorable position in a non-standardized way. |
|  | standardized behavior | Player dribbled the opponent using a simple side touch with the dominant limb. |
|  | non-standardized behavior | Consider all executions that are different from the standardized dribble (i.e., twist off, roulette, double cut, inside out, scissor, dribble “L” or “V”, pull push, outside cut, high wave, 180º spin, slap step on, step kick, U-turn, pull over back, lift above, and others). |
| Shot | successful | An effort to score a goal, with any regular part of the body, which the ball hitting the target or when was required the goalkeeper intervention. |
|  | unsuccessful | All shots off target. |
|  | attempts | A non-standardized off-target shot. |
|  | fluency | Standardized on-target shot (the finalization is effective). |
|  | versatility | Non-standardized on-target shot (the finalization is effective and different). |
|  | standardized behavior | Off-target and on-target shot with the dominant limb, in which the player hitted the ball with the instep or on the top of the foot and also with the inside of the foot. |
|  | non-standardized behavior | Consider all executions or variations that were different from standardized shot (i.e., long distance shooting with ball bouncing, falling shot, high ball first touch, head shot, bicycle shot, and others). |
| Other actions | | Other actions that are not included on the table. |
| Ball possession | | The ball possession is considered when a player respect at least, one of the following assumptions: i) Performs three consecutive contacts with the ball; ii) performs a successful pass (allowing the team to maintain possession of the ball); iii) performs a shot on or off target (finalization). |
