# Supplementary material for: Effects of the Skills4Genius sports-based training program in creative behavior
Source: PLoS One. 2017 Feb 23;12(2):e0172520. doi: 10.1371/journal.pone.0172520 (PMC5322953; doi:10.1371/journal.pone.0172520)
Supplement: S2 Table — (DOCX) [file pone.0172520.s003.docx]

**S2 Table**

Further, it was provided in the S2 table a simplified example of the CBATS structure to measure the technical skill of one player in an ecological situation. Then, the information was organized in the CBATS spreadsheet to carry on the calculations of the attempts, fluency and versatility of technical performance. Thus, the sum of fluent actions for the pass, dribble and shot result in the total score of fluency and was the same process with attempts and versatility. Moreover, the previous variables were assessed considering absolute values of occurrence (frequency).

**S2 Table.** A simplified example of the measurement the in-game technical skills of one player using the Creative Behavior Assessment in Team Sports – CBATS.

| **Creative Behavior Assessment in Team Sports – CBATS** (in ball possession) | | | | | | | | | | | |
| --- | --- | --- | --- | --- | --- | --- | --- | --- | --- | --- | --- |
| **Ball Possession** | **Pass-on- ball** | | | **Dribble** | | | **Shot** | | | **Other Actions** | **Description of the versatile actions** |
|  | unsuccessful | successful | | unsuccessful | successful | | unsuccessful | successful | |  |  |
|  | attempts | fluency | versatility* | attempts | fluency | versatility* | attempts-off target | fluency on-target | versatility on-target* |  |  |
| **1** | 1 | 2 | 0 | 0 | 0 | 0 | 0 | 0 | 0 | 0 | - |
| **2** | 0 | 1 | 0 | 1 | 0 | 0 | 0 | 0 | 0 | 0 | - |
| **3** | 0 | 2 | 1 | 0 | 1 | 0 | 0 | 1 | 0 | 0 | pass with head |
| **4** | 1 | 3 | 0 | 0 | 0 | 1 | 1 | 0 | 0 | 0 | dribble “L” |
| **5** | 0 | 1 | 0 | 0 | 2 | 0 | 1 | 0 | 0 | 0 | - |
| **Score per skill** | 2 | 9 | 1 | 1 | 3 | 1 | 2 | 1 | 0 | 0 | 0 |
|  | | | | | | | | | | | |
| **Total Score** (frequency) | **5** | **13** | **2** |  | | | | | |  |  |
|  | **20** |  |  |  | | | | | |  |  |
